# Book Review

**DOI:** 10.3889/oamjms.2016.031

**Published:** 2016-02-29

**Authors:** Doncho Donev

**Affiliations:** Faculty of Medicine, Ss Cyril and Methodius University of Skopje, Skopje, Republic of Macedonia

**Keywords:** Scientific publishing, publication strategy, publication ethics

## Abstract

**PURPOSE:**

This book provides step-by-step guidance on developing a sound publication strategy for how to prepare and get research papers published. The book is a user-friendly guide, a route map for publishing that covers many topics, ranging from abstracts and blogs, tables and trial registration to ethical principles and conventions for writing scientific papers. Publishing the results of scientific research in the form of a scientific paper is the ultimate goal and the final stage of the research of each scientist. To write and publish papers is never going to be an easy task. With this book as their guide, researchers will be better informed and therefore should have an easier and altogether more pleasant path to publication with clear direction on how to choose the right journal, avoid publication delays, and resolve authorship disputes and many other problems associated with scientific publishing.

**CONTENTS:**

The 188 pages of the book are distributed in 5 chapters in Part I and 249 entries ordered by the letters of Alphabet in Part II creating an A to Z of publication strategy. In the Appendices there are four sections covering further reading, organizations, guidelines and principles of good publication practice for company-sponsored medical research. The book also contains key references and useful websites within many entries where it seemed helpful. The last ten pages of the book present an index to help users to find the information of interest in the book.

**CONCLUSION:**

The book is intended to help all authors, young and old, novice and experienced, to plan their research and publications effectively and prepare manuscripts for journals and other publications, increasing the likelihood that their work will be published. Providing essential information on publishing strategy and process, the book should be extremely useful to everyone who wants to publish research results.

## Field of medicine

Scientific publishing in biomedicine

## Audience

This lively and intelligent guide is primarily directed towards scientific or health-care professionals, students at all levels, medical and other professionals in biomedicine interested in publishing scientific results and observations from their professional work and experience. In particular, it is written for those who intend to carry out scientific research, especially young doctors and healthcare professionals carrying on specialization and students at all levels, from undergraduate to postgraduate. It is applicable to professionals in various clinical disciplines and public health who are involved in writing a research proposal, selecting a proper research strategy, conducting the research itself and submitting a final report. The book can also be useful as a guideline for all medical and other professionals in biomedicine in conducting and promoting their professional and research work by publishing papers in scientific journals.

## Purpose

This book presents an original effort to summarize the relevant data and information about scientific publishing, obtained from authentic and highly regarded sources. It provides step-by-step guidance on developing a sound publication strategy and on the art and science of getting research papers published. The book is user-friendly and an easy-to-read-and-use guide, a route map for publishing that covers many topics, ranging from abstracts and blogs, tables and trial registration to ethical principles for writing scientific papers, conventions and often unwritten rules of publishing in peer-reviewed journals and proceedings at conferences.

In the professional and academic environment it is necessary to continuously publish new findings and results of professional, research and academic work in order to achieve status and career advancement. Proper communication of scientific knowledge, ideas and new scientific discoveries, and knowing how to communicate in this technology-driven world for publishing papers in peer review journals is critically important. Publishing the results of scientific research in the form of a report (i.e. a scientific paper) is the ultimate goal and the final stage of the research of each scientist. The published scientific paper should be a reliable reference and lasting legacy, although it should always remain subject to review and criticism.

To write and publish papers is never going to be an easy task. It requires from scientists a sincere desire for knowledge, imagination and creativity, perseverance in the hard work and writing skills to prepare a scientific paper in accordance with internationally accepted principles and criteria for scientific communication. To write for journals and for other publications is mainly a learned activity and much more science than art. Good writing and producing a well-written article is learned through reading, imitation, repetition, and instruction. With this book as their guide, researchers will be better informed and therefore should have an easier and altogether more pleasant path to publication with clear direction on how to choose the right journal, avoid publication delays, and resolve authorship disputes and many other problems associated with scientific publishing.

## Content

The 188 pages of the book are distributed in 5 chapters in Part I and 249 entries ordered by the letters of Alphabet in Part II creating an A to Z of publication strategy. In the Appendices there are four sections covering further reading, organizations, guidelines and principles of good publication practice for company-sponsored medical research (from GPP3). The book also contains key references and useful websites within many entries where it seemed helpful. The last ten pages of the book present an index to help users to find the information of interest in the book.

## Highlights

The A to Z glossary format used to describe publication strategy is conceptualized upon contemporary knowledge, methods and methodological approaches in science and publishing. This format makes the relevant information easy to look for and easily accessible to readers with varying levels of publication experience and from different backgrounds. The book is a useful tool for individual authors or groups of 2-3 collaborators to understand the publication process in writing up their own small research projects and to avoid common pitfalls that often delay or prevent publication. It is also a helpful guide for authors of large-scale multicentre trials and explains the fundamentals of writing and publishing articles following the IMRAD structure. Furthermore, for undergraduate and postgraduate students and healthcare professionals with little or no experience of publishing, and for those who don’t know what they don’t know, the author has included an overview at the beginning of the book which contains a few longer sections to set the scene.

The publication reflects the rich scientific knowledge of the author as an active researcher, writer, teacher and advocate, with more than 20 years experience in publishing, teaching, editing and coordinating publications for drug companies and individual researchers. Her recommendations are evidence based where possible, and qualified accordingly when evidence is lacking.

## Conclusion

The book should help all authors, young and old, novice and experienced, and even those who don’t know what they don’t know, to plan their research and publications effectively and prepare manuscripts for journals and other publications, increasing the likelihood that their work will be published. Providing essential information on publishing strategy and process, the book should be extremely useful, and I warmly and thoroughly recommend it to everyone who wants to publish research results. The book is reasonably priced and can be ordered from the publisher (CRC Press Taylor & Francis Group) at:

https://www.crcpress.com/Getting-Research-Published-An-A-Z-of-Publication-Strategy-Third-Edition/Wager/9781785231384?promo=LBP20

